# MRMaid: The SRM Assay Design Tool for *Arabidopsis* and Other Species

**DOI:** 10.3389/fpls.2012.00164

**Published:** 2012-07-20

**Authors:** Jun Fan, Fady Mohareb, Alexandra M. E. Jones, Conrad Bessant

**Affiliations:** ^1^Cranfield Health, Cranfield Bioinformatics Group, Cranfield UniversityBedfordshire, UK; ^2^The Sainsbury Laboratory, Norwich Research ParkNorwich, UK

**Keywords:** selected reaction monitoring, multiple reaction monitoring, proteomics, transition, database, *Arabidopsis*, experimental design

## Abstract

Selected reaction monitoring (SRM), sometimes called multiple reaction monitoring (MRM), is becoming the tool of choice for targeted quantitative proteomics in the plant science community. Key to a successful SRM experiment is prior identification of the distinct peptides for the proteins of interest and the determination of the so-called *transitions* that can be programmed into an LC-MS/MS to monitor those peptides. The transition for a given peptide comprises the intact peptide *m*/*z* and a high intensity product ion that can be monitored at a characteristic retention time (RT). To aid the design of SRM experiments, several online tools and databases have been produced to help researchers select transitions for their proteins of interest, but many of these tools are limited to the most popular model organisms such as human, yeast, and mouse or require the experimental acquisition of local spectral libraries. In this paper we present MRMaid^1^, a web-based SRM assay design tool whose transitions are generated by mining the millions of identified peptide spectra held in the EBI’s PRIDE database. By using data from this large public repository, MRMaid is able to cover a wide range of species that can increase as the coverage of PRIDE grows. In this paper MRMaid transitions for 25 *Arabidopsis thaliana* proteins are evaluated experimentally, and found capable of quantifying 23 of these proteins. This performance was found to be comparable with the more time consuming approach of designing transitions using locally acquired orbitrap data, indicating that MRMaid is a valuable tool for targeted *Arabidopsis* proteomics.

## Introduction to Selected Reaction Monitoring

In recent years, selected reaction monitoring (SRM) has become the method of choice for targeted quantitative plant proteomics. The attraction of SRM is twofold (Anderson and Hunter, 2006; Lange et al., 2008). Firstly, it is possible to perform SRM on a relatively inexpensive triple quadrupole (QQQ) instrument coupled to liquid chromatography (LC). Secondly, the ultimate sensitivity of SRM is much higher than traditional shotgun proteomics. This higher sensitivity is achieved by pre-programming the QQQ to monitor specific tryptic peptides that represent proteins of interest (so-called *proteotypic* peptides). By focusing on the *m/z* of these peptides and their most reliable product ions at the retention times (RTs) during which they are expected to emerge from the LC, sensitivity is greatly increased compared to shotgun proteomics which requires the mass spectrometer (MS) to scan across the whole mass range and only identifies peptides with the highest precursor intensities. Performing targeted analysis in this way on a QQQ substantially increases signal to noise ratio compared to the traditional shotgun approach.

A key prerequisite to any successful SRM experiment is selection of an appropriate set of peptides and product ions to monitor for the proteins of interest. As a minimum, for each protein it will be necessary to identify a peptide which does not map to anywhere else within the proteome of the species under study. However, it is well known that some peptides cannot be detected by mass spectrometry due to poor ionization and other factors, so it is important to ensure the selected peptide is detectable. Since some peptides may share the same precursor *m/z* and RT it is also necessary to select at least one product ion which is specific to the selected peptide, so that this can be monitored in the third quadrupole. The key criteria for selecting the product ion are that it should produce a reliable, high intensity peak that is significant compared to any surrounding spectral noise. The specific combination of peptide *m/z* and *m/z* of a reliable product ion is referred to as a transition and it is this transition which is used to target the protein of interest at a specific RT.

It is good practice to select multiple peptides per protein and several product ions per peptide so that quantitative precision can be maximized and errors estimated. This may involve designing as many as 20 transitions per protein (e.g., four peptides with five product ions each), and in most experiments there is more than one protein of interest so the number of transitions required for a given experiment can easily run into the hundreds. Designing this many transitions manually is extremely time consuming, so bioinformaticians have devised software to assist in the process (Cham Mead et al., 2010). Here we present a web-based tool, called MRMaid, which uses proteomic spectra held with the EBI’s PRIDE database (Vizcaino et al., 2009) to produce lists of recommended SRM transitions for lists of proteins entered by the user.

## The MRMaid SRM Assay Design Tool

### Using MRMaid

MRMaid is a freely available web-based tool accessible at www.mrmaid.info. On opening the web page, the user is presented with a simple search page. Within this search page is a drop down list from which the species under study can be selected, and a text box in which the protein, or proteins, of interest must be entered. Proteins can be specified using their accession numbers or protein names. MRMaid uses PICR (Côté et al., 2007) to map the entered accession numbers to the SwissProt identifiers that it uses internally, so many different types of accession are supported, including AGI. Multiple proteins may be entered, separated using spaces, commas, or tabs. The transition list for the protein(s) of interest can then be retrieved by clicking the “MRMaid Search” button.

MRMaid returns the transition list in the form of a large table, in which each row represents an individual transition (a specific product ion for a particular peptide related to a protein that was entered in the search box). The transitions shown are those that MRMaid considers to be the best available for the specified proteins, based on experimental evidence held in the PRIDE database (details of how the transitions are derived from PRIDE are provided in the next section). The number of transitions shown in the table can be modified by specifying the maximum number of peptides per protein and product ions per peptide in the drop down boxes above the table. There is also a drop down box for filtering the results according the type of instrument on which the experimental evidence was acquired.

The columns of the transition table carry information about each transition, information that is either identification data (e.g., protein name, peptide sequence, product ion name), transition data (i.e., precursor *m/z*, RT and product ion *m/z*), or metrics used to rank the transitions. The information reported for each transition is summarized in Table 1. The transition table is fully interactive, so clicking on a column heading sorts the transitions in order according to the values in that column. Transitions may also be filtered according to peptide sequence, peptide score, and observation metrics by entering filtering criteria into the boxes under the relevant column headings.

**Table 1:** **Description of transition characteristics shown in MRMaid’s transition table**.

Column heading	Description
Protein	The protein name or accession number to which the transition relates
Sequence	Sequence of the recommended peptide, together with flanking amino acids
*P*_peptide_	Probability of observing the suggested peptide when the parent protein is present, based on identification evidence in PRIDE
Peptide score	A measure of the suitability of a peptide for SRM, primarily based on the quality of fragmentation spectra held in PRIDE for the peptide, using the algorithm described in Mead et al. (2009)
RT	Estimated retention time based on the peptide sequence, calculated using the SSRCalc algorithm (Krokhin et al., 2004). The parameters used to calculate RT can be set for a particular LC setup using the “HPLC Conditions” parameters above the table
Production	The name of the product ion to monitor, e.g., y8
Production observation	The number of times the product ion has been seen in PRIDE for this peptide
*P*_product_	Probability of observing the specified product ion when the parent peptide is observed – as calculated across all relevant PRIDE experiments
Precursor charge	List of observed precursor charge states, with their frequency of occurrence in brackets
Precursor *m/z*	List of precursor *m/z* to monitor for the suggested peptide – one for each observed charge state
Production *m/z*	The product ion *m/z* window to monitor for the suggested ion
Production relative intensity	Average intensity of the ion, relative to the most intense ion in each fragmentation spectrum, calculated across all relevant PRIDE spectra
PRIDE data	The number of PRIDE experiments in which the peptide has been seen. Clicking on a number in this column brings up a list of PRIDE experiments, and clicking on one of these opens the relevant experiment in PRIDE

Having manipulated the transition table to show preferred transitions, those to be exported for use in the laboratory can then be selected by clicking the relevant check boxes in the leftmost column of the table. The selected transitions can then be saved to a local file by clicking an “Export” button at the foot of the table. MRMaid thereby provides a simple way to retrieve, organize, and select transitions for a specified collection of proteins, and provides these transitions in a file that can be used in the laboratory.

### MRMaid transition design

**Figure 1:**
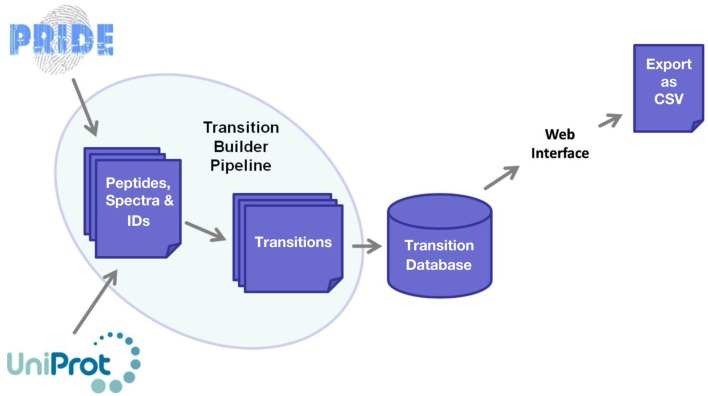
**Software framework underlying MRMaid**. The core of the system is the transition database, from which transitions for proteins of interest are extracted and displayed to users via the web interface. The transition database is populated by MRMaid’s transition builder pipeline, which takes SwissProt protein sequences from UniProt and mines spectral data in PRIDE to identify the best transitions for each protein.

The software framework underpinning MRMaid is shown in Figure 1. The core of the system is the transition database. This database contains all of MRMaid’s transitions (currently a total of 473,373 for *Arabidopsis* alone), and it is from here that the transitions are retrieved for display in the transition table when a search is performed via the web interface. All the transitions are pre-calculated, allowing results to be returned to the user in an acceptable time frame regardless of the amount of experimental evidence underlying each transition.

The transition database is populated by the transition builder pipeline, shown on the left of Figure 1. The starting point for this pipeline is the SwissProt proteome for the species being studied (UniProt Consortium, 2011). From this proteome, all possible tryptic peptides are calculated for each protein and then checked against the rest of the proteome for sequence uniqueness. Since non-unique peptides cannot be used for SRM, only unique peptides are retained for subsequent steps in the pipeline.

For each unique peptide, all available spectral data and associated metadata is retrieved from the EBI’s PRIDE database. PRIDE is a major repository containing a rapidly growing and increasingly high quality body of proteomics data. At the time of writing, PRIDE holds nearly 300 million spectra (of which 51 million have been matched to peptides) from over 100 different species, with seven million spectra for *Arabidopsis* alone. The principle behind MRMaid is that this immense amount of experimental evidence can be used to determine how suitable each unique tryptic peptide may be for SRM, and which product ions are the best to monitor for that peptide. Since not all PRIDE data is curated, it is necessary to perform some data quality filtering but this only removes a minority of the available data for most peptides.

The next step in the transition builder pipeline process is to calculate transition metrics for each peptide based on the available data. As mentioned in Section “Introduction to Selected Reaction Monitoring,” detectability of the peptide is of utmost importance. This is calculated from the PRIDE data as a simple ratio of the number of times the peptide was observed to the number of times its parent protein was observed. This ratio, reported by MRMaid as *P*_peptide_, provides a simple measure of a peptide’s detectability – the higher the ratio the better the peptide is for SRM.

The second transition metric calculated is the *peptide score*, which takes into account a number of characteristics of the spectral data found in PRIDE. SRM practitioners typically target *y* ions in the product ion spectrum, so these characteristics include the number of observed *y* ions and their abundance in relation to other spectral peaks. The principles behind the peptide score calculation are described in a previous paper (Mead et al., 2009), when the peptide score was referred to as the *transition score*.

Finally, the transition builder pipeline seeks to rank individual product ions according to their potential suitability for monitoring in SRM. This is achieved by calculating a simple observability ratio, *P*_product_, from all the spectra available for each peptide. The product ion intensity across all spectra for that peptide is also calculated. Product ions that are frequently observed when their parent peptide is present and generally produce high intensity peaks are clearly good candidates for SRM.

Following the steps above, the transition builder pipeline picks out relevant peptides and their corresponding product ions and deposits these in the MRMaid transition database as possible transitions along with the aforementioned transition metrics. This process can take several days per species so is carried out offline, separately from the MRMaid web server. The process is repeated periodically to ensure that the latest PRIDE data is considered.

## MRMaid Assay Validation

Although MRMaid’s transitions are derived from experimental data, ultimately they are still predictions so are not guaranteed to work in the laboratory. To evaluate how good the predictions are, a validation experiment was conducted in which a substantial list of transitions produced by MRMaid for *Arabidopsis* proteins were tested experimentally.

### Validation materials and methods

#### Preparation of sample and selection of proteins

Total soluble proteins were extracted from *Arabidopsis thaliana* Col0 cell culture, as described in Jones et al. (2009) and stored in 80% acetone at −20°C. Proteins were solubilized in 8 M urea after removal of the acetone. Reduction with 50 mM DTT was performed at room temperature for 1 h before alkylation with 200 mM iodoacetamide (1 h, room temperature in the dark). The mixture was diluted with 100 mM ammonium bicarbonate to bring the urea concentration to ∼ 2 M before the addition of trypsin (Promega) at a ratio of 1:100. The proteins were digested overnight at 37°C before the addition of acetic acid and storage at −20°C. The digest was simply diluted 10-fold with 5% formic acid before analysis by mass spectrometry.

The mixture of proteins from a total cell digest was expected to be very complex. To determine which proteins were present in the sample at a detectable level – and thereby provide a fair test of MRMaid’s predicted transitions – a small portion was analyzed using LC and tandem mass spectrometry (Orbitrap XL, Thermo Scientific) as described in Greer et al. (2012) with the difference that only 2^+^ or 3^+^ ions were selected for data dependent fragmentation. Peak lists were extracted from Thermo Scientific’s “RAW” files using msconvert (proteowizard.sourceforge.net) in mzML format and searched against the *Arabidopsis* genome (TAIR v10) using Mascot (v 2.3.02, Matrix Science). For the Mascot search precursor masses were allowed 10 ppm (monoisotopic) error, fragments 0.6 Da and +57 (Carbamidomethyl) was specified as a fixed modification on cysteine residues and +16 (oxidation) as a variable modification on methionine resides and two tryptic mis-cleavages were allowed. The Mascot dat file was further parsed by Scaffold (v 3.3.3 Proteome Software). To be accepted as a valid protein identification two peptides at 95% probability were required as a minimum, giving 0.1% FDR at the protein level. Over 100 proteins were identified (Table S1 in Supplementary Material) and the top 26 SwissProt proteins, ranked by number of unique peptides, were selected as candidate proteins to test MRMaid.

#### Generation of MRMaid transition list

The *Arabidopsis* AGI locus identifiers (TAIR) were converted to SwissProt/Trembl identifiers using the Uniprot mapping tool^2^ (Table S2 in Supplementary Material). To provide a reasonable set of transitions to test, four peptides and five transitions were requested from MRMaid for each protein. MRMaid output is presented in Table S3 in Supplementary Material. To create a simplified tab separated file, compatible with MassLynx software (Waters) that runs the TQ-S MS, we created a simplified list by manual editing to choose charge state with most previous observations and checking that the m/z and charge state was the same for all transitions of the peptide (Table S4 in Supplementary Material). Since RT prediction was not the focus of this study, no RTs were specified and the transitions were split into three methods to provide adequate MS cycle and dwell times (minimum 3 ms per transition). With a typical LC peak width of >20 s this provides at least 15 points per peak.

#### Testing predicted transitions

The peptides were analyzed using nano-spray ESI and a TQ-S MS (Waters Corp., MA, USA). The LC system consisted of a nanoAcquity with a Symmetry trap (Waters, C18, 180 μm × 20 mm) to concentrate and desalt the peptides before elution to the analytical column (Waters, BEH 100 mm C18 columns, 75 μm i.d., 1.7 μm beads) a flow rate of 400 nl/min was used with a gradient from 5% acetonitrile to 35% acetonitrile over 40 min. Two replicate injections were performed.

The resultant TQ-S files were imported into Skyline (MacLean et al., 2010) and the peak definitions checked manually. The peak areas were then exported into Excel (Microsoft) for further analysis.

#### Using in-house data with skyline

To test the performance of MRMaid and the data available in PRIDE against the use of in-house data, we used the fragmentation data acquired on the Orbitrap (see Preparation of Sample and Selection of Proteins) and the program Skyline to design an alternative set of transitions, using the same proteins and requiring four peptides per protein with five transitions. Two missed cleavages were allowed, and peptides were selected based on their spectrum count in the orbitrap data. Transitions were allowed to be *b* or *y* ions and selected based on their intensity (Table S5 in Supplementary Material). Collision energy was the default recommended for “Waters Xevo” in Skyline and used without further optimization. The total number of transitions was 495, for 99 precursors, these transitions were divided into two methods and analyzed on the same LC-MS system as described in Section “Testing Predicted Transitions.”

### Validation results and discussion

#### Coverage of MRMaid transitions

Of the 26 protein candidates 25 proteins were represented in MRMaid. The missing protein, Q0WL56, had only recently been added to SwissProt, after the most recent execution of MRMaid’s transition builder pipeline so was not present in the transition database build used in this study. We looked for four peptides and five transitions per protein; out of the possible total 500 transitions MRMaid returned 95 peptides with 466 transitions.

Of the 34 missing transitions, 15 are associated with P51818 (heat shock protein 81). This protein contains only a single proteotypic peptide so it is impossible for MRMaid to return the requested four peptides and their product ions. Q9FEF8 (rRNA 2′-*o*-methyltransferase fibrillarin 1) is responsible for a further 10 of the missing transitions. Although this protein contains seven proteotypic peptides, PRIDE only contains sufficient data for two of these. The remaining nine missing transitions are due to MRMaid’s inability to find five product ions for peptides from three of the proteins (P25858, Q9ZRW8, and P60040), due to insufficient data in PRIDE at the present time.

#### Results of MRMaid predicted transitions

A transition was considered to be observed if the peak intensity was greater than 10,000 in both replicate injections, by this measure 343 of MRMaid’s recommended transitions were observed (74%). A peptide was counted as being observed if three or more of its transitions were observed, of the 95 peptides for which we designed transitions, 68 were counted (72%). At the protein level, we set a threshold of two peptides observed with three or more transitions – a common minimum requirement. By this measure MRMaid performed particularly impressively with 23 out of 25 (92%) of proteins returning at least two observable peptides. Of the two unobserved proteins, the first was P51818, which could not meet the two peptide criterion because it has only one proteotypic peptide (even though four of the five recommended transitions were observed for this peptide). The second protein, Q9MAH0 (PPC1 phosphoenolpyruvate carboxylase), also had a single observed peptide, despite four peptides each with five transitions being returned by MRMaid. The results are summarized in Table 2 and presented in full in Table S6 in Supplementary Material.

**Table 2:** **Numbers of designed and observed transitions from MRMaid and Skyline**.

	MRMaid from PRIDE data		Skyline from local orbitrap data	
	Designed	Observed	Designed	Observed
Proteins	25	23	25	24
Peptides	95	68	99	79
Transitions	466	343	495	421

*Although Skyline performs slightly better, the criteria used for selecting peptides in Skyline were much less conservative than those used in MRMaid*.

#### Results of orbitrap/skyline transitions

Overall the Orbitrap/Skyline combination performed only slightly better than MRMaid returning usable transitions for 24 proteins (including P51818 as peptide uniqueness was not set as a requirement in Skyline), 79 peptides, and 421 transitions (Table 2, Table S6 in Supplementary Material). However this result is striking because the difference is relatively small and one might assume that the in-house database would have the advantage because the peptides selected for SRM were already positively identified as being present in the sample. Notably there was one protein, O50008, for which the Orbitrap/Skyline predictions gave no usable peptide transitions, whereas MRMaid returned two usable peptides, with a total of nine transitions between them.

Where the programs selected the same peptides, generally the same transitions were selected. When different peptides from the same protein were selected, often the resulting intensities were comparable, especially if summed over the protein. Overall peptides selected by either program showed a clear tendency to “succeed” where all five transitions were observed or “fail” where no transitions were seen. This indicates an inability to detect at the peptide level, for which there are many possible explanations. For both MRMaid and Skyline transitions there appeared to be some correlation between larger precursor *m/z* and inability to observe transitions. We had generally favored the doubly charged form when selecting precursor masses. To test if charge state had a significant impact on the success of the transition we selected 25 peptides with an *m/z* greater than 1000 for the 2^+^ ion and tested these in a single run with both 2^+^ and 3^+^ charge states (Table S6 in Supplementary Material). Generally transitions from larger peptides performed better with triply charged precursors (16/25), as judged by peak intensity and four performed better as 2^+^, for one peptide the intensity was equal between charge states and four peptides could not be detected in either charge state. We therefore recommend that both common charge states should be assayed when optimizing methods.

## Validation Discussion

Overall there appeared to be a good representation of proteins in the current build of MRMaid as transitions were returned for 25 of the 26 targeted proteins. The missing protein will be covered in the near future, as the MRMaid transition database is periodically rebuilt using the latest SwissProt and PRIDE databases. Further analysis shows that the current MRMaid build contains transitions for 7,165 *Arabidopsis* proteins, from the 11,018 proteins available in SwissProt. We concede that SwissProt is conservative and that greater coverage would be achieved if proteomes from TrEMBL or TAIR were used instead.

The peptides and transitions returned by MRMaid performed as well as the alternative solution of building an in-house spectral library using Skyline. As discovery MS platforms are expensive and generating discovery data can be demanding on both time and resources, MRMaid provides a valuable alternative by using publically available data instead. It should be noted that MRMaid is more conservative than our use of Skyline as non-unique, modified, or miscleaved peptides are currently not considered by MRMaid as suitable SRM candidates, whereas all these were permitted in Skyline. This is the main factor in the difference in coverage between MRMaid and Skyline.

## Conclusion and Future Plans

MRMaid is a useful tool for SRM assay design. It is easy to use thanks to a simple and intuitive web-based interface which requires no software or data to be downloaded to the user’s machine. Testing MRMaid’s transitions on *Arabidopsis* protein extracts in the laboratory using a triple quadrupole MS showed that the majority (72%) of recommended peptides were detectable and that a similarly high proportion (74%) of product ions recommended produced high intensity peaks that could be used for quantitation. Collectively, this provided sufficient information to confidently quantify 92% of the proteins of interest. This confirms that it is possible to use ESI-trap data from PRIDE as the basis for designing SRM assays for triple quadrupole instruments. *Arabidopsis* researchers can therefore have confidence in SRM assays produced by MRMaid.

In terms of future plans, we are committed to extending the coverage of MRMaid beyond *Arabidopsis* to further species for which there is sufficient spectral data in PRIDE. The next plant in our schedule is *Zea mays*, for which PRIDE holds 14 million spectra at the time of writing. Clearly, the scope for supporting further species is totally dependent on the available data in PRIDE, so we would strongly encourage submission of plant proteomic data to PRIDE. We will also be adding the option to export transition lists in the recently released HUPO PSI TraML standard data format (Deutsch et al., 2012), to facilitate more direct transfer of MRMaid’s transition lists to laboratory instruments.

## Conflict of Interest Statement

The authors declare that the research was conducted in the absence of any commercial or financial relationships that could be construed as a potential conflict of interest.

## Supplementary Material

Supplementary Material for this article can be found online at http://www.frontiersin.org/Plant_Proteomics/10.3389/fpls.2012.00164/abstract

Table S1 Proteins identified from discovery Orbitrap LC-MS analysis to generate candidate protein list.

Table S2 List of candidate proteins for testing MRMaid.

Table S3 MRMaid output for 25 candidate proteins.

Table S4 Simplified transition list manually edited from MRMaid output.

Table S5 Transition list derived from Orbitrap discovery LC-MS analysis and Skyline.

Table S6 Full results from PRIDE-MRMaid predicted transitions, Orbitrap-Skyline transitions and testing of 2+ or 3+ charge states for peptides over 1000 *m*/*z*.

## Footnotes

^2^http://www.uniprot.org/mapping/
